# Anti-Inflammatory Effects of *Licania macrocarpa* Cuatrec Methanol Extract Target Src- and TAK1-Mediated Pathways

**DOI:** 10.1155/2019/4873870

**Published:** 2019-09-12

**Authors:** Kon Kuk Shin, Jae Gwang Park, Yo Han Hong, Nur Aziz, Sang Hee Park, Sunggyu Kim, Eunji Kim, Jae Youl Cho

**Affiliations:** ^1^Department of Integrative Biotechnology, Sungkyunkwan University, Suwon 16419, Republic of Korea; ^2^Division of Translational Science, Research Institute, National Cancer Center, Goyang 10408, Republic of Korea; ^3^Department of Biocosmetics, Sungkyunkwan University, Suwon 16419, Republic of Korea; ^4^Research and Business Foundation, Sungkyunkwan University, Suwon 16419, Republic of Korea

## Abstract

In this study, we investigated the anti-inflammatory effects of *Licania macrocarpa* Cuatrec methanol extract (Lm-ME) in vitro and in vivo and found pharmacological target proteins of Lm-ME in TLR4-mediated inflammatory signaling. This extract reduced NO production and mRNA expression of inflammatory cytokines such as iNOS, COX-2, IL-6, and IL-1*β*. In the NF-*κ*B- and AP-1-mediated luciferase reporter gene assay, transcription factor activities decreased under cotransfection with MyD88 or TRIF. Phosphorylated protein levels of Src, PI3K, IKK*α*/*β*, and I*κ*B*α* as well as p50 and p65 in the NF-*κ*B signal pathway were downregulated, and phosphorylation of TAK1, MEK1/2, MKK4/7, and MKK3/6 as well as ERK, JNK, and p38 was decreased in the AP-1 signal pathway. Through overexpression of HA-Src and HA-TAK1, respectively, Lm-ME inhibited autophosphorylation of overexpressed proteins and thereby activated fewer downstream signaling molecules. Lm-ME also attenuated stomach ulcers in an HCl/EtOH-induced acute gastritis model mice, and COX-2 mRNA expression and phosphorylated TAK1 levels in gastric tissues were diminished. The flavonoids kaempferol and quercetin were identified in the HPLC analysis of Lm-ME; both are actively anti-inflammatory. Therefore, these results suggest that Lm-ME can be used for anti-inflammatory remedy by targeting Src and TAK1.

## 1. Introduction

Inflammation is the first defense against many infectious pathogens or injury, and it plays an important role in common causes of atherosclerotic cardiovascular disease, cancer, and chronic obstructive lung disease [1–3]. In particular, toll-like receptors (TLRs) are key factors of the innate immune system that also recognize pathogen-associated molecular patterns (PAMPs), which are molecular motifs shared by different types of microbes [4, 5]. TLRs are expressed on dendritic cells, macrophages, natural killer cells, T cells, B cells, epithelial cells, endothelial cells, and fibroblasts [6]. Four particular adapter molecules of TLRs are known to be involved in signaling: MyD88, TIRAP, TRIF, and TRAM [7].

TLR4 recognizes microbial components, including lipopolysaccharides (LPS) and saturated fatty acids (SFA) [8], and can induce signaling through MyD88 and TRIF [9]. In the case of TLR4 activation, there are two different ways to activate inflammation: a MyD88-dependent pathway and a MyD88-independent pathway. In the case of the MyD88-dependent pathway, MyD88 recruits IRAK4 to activate and degrade IRAK1. IRAK1 is associated with TRAF6, and the complex can activate TAK1, which activates the I*κ*B kinase (IKK) and mitogen-activated protein kinase (MAPK). This IKK-related complex can activate NF-*κ*B, and the MAPK-related protein activates the AP-1 pathway [8, 10]. In the MyD88-independent pathway, however, TRIF takes part in late-phase NF-*κ*B and MAPK signaling, because TRIF recruits TRAF6 and receptor-interacting protein 1 (RIP1) to activate TAK1, resulting in NF-*κ*B activation. Additional TLR4 downstream signaling molecules PI3K and AKT also activate the NF-*κ*B pathway [11–13].

NF-*κ*B and AP-1 transcription factors can induce inflammatory cytokines such as TNF-*α*, IL-1*β*, IL-6, iNOS, and COX-2 [14, 15]. These cytokines can induce pathological pain and promote inflammation [16]. Nitric oxide (NO), in particular, is produced by inducible nitric oxide synthase (iNOS) and plays roles in blood pressure regulation, inflammation, infection, and the onset of progression of malignant disease [17]. Poor regulation of these inflammation symptoms can lead to many diseases, including cardiovascular disease, cancer, rheumatoid arthritis, allergy, asthma, and Alzheimer's and chronic kidney disease [18]. Accordingly, many researchers have focused on inflammatory signaling targets and also identified candidate substances that prevent inflammatory cytokine production and inflammation-related protein or gene expression.

*Licania macrocarpa* Cuatrec belongs to the genus *Licania* (chrysobalanaceae), which consists of more than 200 species distributed mainly in the Americas, especially from Panama to Peru [19]. *Licania* species have been used as medicinal plants in South America to treat inflammation, diabetes, stomach ailments, diarrhea, and dysentery [20]. Despite traditional use, the medicinal mechanisms of the genus *Licania* have not been well characterized at the cellular and molecular levels. Therefore, we focused on the anti-inflammatory effects of a methanol extract of *L. macrocarpa* Cuatrec (Lm-ME) both in vitro, by using macrophage-like RAW264.7 cells, and in vivo, by using an HCl/EtOH-induced acute gastritis mouse model. To check for anti-inflammatory effects, we conducted an NO assay in macrophage-like RAW264.7 cells and peritoneal macrophages and also examined inflammation-related mRNA expression of cytokines such as IL-6, IL-1*β*, iNOS, and COX-2. To find target proteins that are affected by Lm-ME in the TLR4 pathway, we utilized a luciferase reporter gene assay, western blotting, and an overexpression strategy. To identify the active components of Lm-ME, we carried out HPLC analysis using flavonoids related to anti-inflammatory effects.

## 2. Materials and Methods

### 2.1. Materials

Methanol extract of *L. macrocarpa* Cuatrec (Lm-ME; code no: PBEC10204) was purchased from the Plant Extract Bank of the Plant Diversity Research Centre (Daejeon, Korea). RAW264.7 cells (a BALB/c-derived murine macrophage cell line (ATCC No. TIB-71)) and HEK293T cells (a human embryonic kidney cell line (ATCC No. CRL-1573)) were purchased from ATCC (Rockville, MD, USA). Cell culture reagents such as media (RPMI 1640 and DMEM) and FBS were purchased from Hyclone (Grand Island, NY, USA) and Biotechnics Research (Lake Forest, CA, USA). Dimethylsulfoxide (DMSO), 3-(4,5-Dimethylthiazol-2-yl)-2,5-diphenyltetrazolium bromide (MTT), lipopolysaccharide (LPS, Escherichia coli 0111:B4), N^*ω*^-nitro-L-arginine methyl ester hydrochloride (L-NAME), quercetin, kaempferol, luteolin, carboxymethylcellulose (CMC), PP2, and 5Z-7-Oxozeaenol were purchased from Sigma Chemical Co. (St. Louis, MO, USA). Luciferase constructs containing NF-*κ*B- or AP-1-binding promoters were acquired from Promega (Madison, WI, USA). Epitope-tagged constructs (FLAG-MyD88, CFP-TRIF, HA-Src, and HA-TAK1) were obtained from Addgene (Cambridge, MA, USA). Phospho-specific and total antibodies against p50 (Catalog number: #4806, #3035), p65 (#3033, #8242), I*κ*B*α* (#5209, #4812), IKK*α*/*β* (#2697, #2682), Src (#2101, #2109), p85 (#4228, #4292), ERK (#9101, #4696), JNK (#9255, #4672), p38 (#4631, #9212), MEK1/2 (#9121, #9122), MKK4 (#9151, #9152), MKK3/6 (#9236, #9238), MKK7 (#4171, #4172), TAK1 (#9339, #4505), IRAK1 (#4504), IRAK4 (#4363), FLAG (#8146), TRIF (#4596), HA (#5017) and loading control proteins (lamin A/C (#4777), and *β*-actin (#4967)) were purchased from Cell Signaling (Beverly, MA, USA).

### 2.2. Cell Culture

RAW264.7 cells were cultured in RPMI 1640 with 10% heat-inactivated FBS and antibiotics (penicillin and streptomycin). HEK293T cells were cultured in DMEM with 5% heat-inactivated FBS and antibiotics (penicillin and streptomycin). All cells were incubated at 37°C under 5% CO_2_.

### 2.3. Lm-ME Treatment and Administration

A stock of Lm-ME (25.93 g) was dissolved in 100% DMSO (1.10 g/ml) to make 100 mg/ml of Lm-ME for in vitro experiments. Lm-ME was further diluted to 50–200 *μ*g/ml for treatment with medium. For in vivo experiments, the stock of Lm-ME was suspended in 0.5% CMC at a concentration of 100 mg/kg and 200 mg/kg.

### 2.4. NO Production

Macrophage-like RAW264.7 (1 × 10^6^ cells/ml) cells were precultured for 18 h and then pretreated with Lm-ME (0, 50, 100, and 200 *μ*g/ml) for 30 min, followed by LPS (1 *μ*g/ml) treatment for 24 h. In some cases, L-NAME (0.5, 1, and 2 mM), PP2 (10, 20, and 40 *μ*M), and 5Z-7-oxozeaenol (0.625, 1.25, and 2.5 *μ*M) were used for pretreatment instead of Lm-ME. To measure NO production, 100 *μ*l of supernatant of cell culture media and 100 *μ*l of Griess reagent were mixed for 1 min, and absorbance was measured at 540 nm using a spectrophotometer.

### 2.5. Cell Viability Test

The cytotoxic effects of Lm-ME in RAW264.7, HEK293T, and peritoneal macrophage cells were determined by conventional MTT assay as previously described [21].

### 2.6. High-Performance Liquid Chromatography (HPLC)

To identify the active components of Lm-ME, high-performance liquid chromatography (HPLC) was conducted as reported previously [22]. Standard compounds quercetin, kaempferol, and luteolin were used.

### 2.7. Semiquantitative Reverse Transcriptase-Polymerase Chain Reaction (RT-PCR)

RAW264.7 cells were pretreated with 0, 50, 100, or 200 *μ*g/ml of Lm-ME for 30 min and then incubated for 6 h with 1 *μ*g/ml of LPS. Semiquantitative RT-PCR was conducted as described previously [23]. The primer sequences are listed in Table 1.

### 2.8. Luciferase Reporter Gene Assay and Transfection

For the luciferase assay, HEK293T cells (1.25 × 10^5^ cells/well) were plated in 24-well plates for 18 h. Luciferase genes (NF-*κ*B or AP-1), signaling construct (MyD88 or TRIF) and the *β*-galactosidase gene (as a control) were mixed together in Opti-MEM media for 15 min, and the transfection reagent, PEI, was diluted with Opti-MEM for 15 min. After stabilization of the genes and PEI, both were mixed together for 20 min. The mixture was then added to HEK293T cells for 24 h. For transfection of Src and TAK1, HEK293T cells (2.5 × 10^5^ cells/ml) were plated in 6 wells for 18 h. The HA-Src or HA-TAK1 genes were diluted in Opti-MEM, and PEI was also diluted in Opti-MEM, each for 15 min. After stabilization of the genes and PEI, they were mixed together for 20 min. The mixture was added to HEK293T cells for 24 h.

### 2.9. Western Blotting Analysis

Nuclear fraction lysate and total lysates of Lm-ME-treated RAW264.7 (1 × 10^6^ cells/ml) and HEK293T (2.5 × 10^5^ cells/ml) cells were extracted with lysis buffer as described previously [24]. Tissue lysates of HCl/EtOH-induced mouse stomachs were also prepared. Immunoblotting analysis was performed as previously reported [25]. The antibodies against total and phosphorylated forms of IKK*α*/*β* (#2697, #2682), I*κ*B*α* (#5209, #4812), p50 (#4806, #3035), p65 (#3033, #8242), Src (#2101, #2109), p85 (#4228, #4292), ERK (#9101, #4696), JNK (#9255, #4672), p38 (#4631, #9212), MEK1/2 (#9121, #9122), MKK4 (#9151, #9152), MKK3/6 (#9236, #9238), MKK7 (#4171, #4172), TAK1 (#9339, #4505), IRAK1 (#4504), IRAK4 (#4363), *β*-actin (#4967), FLAG (#8146), TRIF (#4596), and HA (#5017) were evaluated.

### 2.10. Preparation of Peritoneal Macrophages

Peritoneal macrophages were collected from peritoneal exudates of ICR mice that were injected intraperitoneally with 4% thioglycollate broth (1 ml, Difco Laboratories, Detroit, MI, USA). After 4 days, the mice were anesthetized with isoflurane and sacrificed. Five ml of PBS was injected into the belly and massaged. After enough massage, PBS was collected into a 50 ml tube 4 times. Cells were pelleted by centrifugation (3,000 rpm, 5 min, 4°C). After discarding the supernatant, the pelleted cells were resuspended and washed with 10 ml of RBC buffer and centrifuged. After lysis of the red blood cells, the remaining cells were resuspended with RPMI media and plated into 96 wells.

### 2.11. HCl/EtOH-Induced Gastritis Mice

Acute gastritis was induced with 300 *μ*l of 150 mM HCl in 60% EtOH as previously reported in ICR mice [14]. We orally injected 0, 100, or 200 mg/kg of Lm-ME for the gastritis-induced groups and 40 mg/kg of ranitidine for the control group 3 times in ICR mice. Eight hour after the last oral injection, 300 *μ*l of 150 mM HCl/60% EtOH was orally administrated for 1 h and ICR mice were anesthetized with isoflurane and sacrificed. The stomachs were excised, rinsed with PBS, and cut along the greater curvature. The area of mucosal erosive lesions was measured by Image J and counting the pixels of lesions.

### 2.12. Real-Time Polymerase Chain Reaction (Real-Time PCR)

The mRNA expression level of gastritis stomach sample was quantified in gastritis stomach samples. mRNA was isolated with TRIzol Reagent (Gibco) according to the manufacturer's manual. mRNA levels were quantified by real-time reverse transcription-polymerase chain reaction using SYBR premix Ex Taq (PCR Biosystems, London, UK). The real-time mRNA expression results were calculated relative to the housekeeping gene GAPDH. The primer sequences are listed in Table 2.

### 2.13. Statistical Analyses

All data presented in this study are expressed as means ± standard deviation (SD) from three independent experiments. For statistical comparisons, ANOVA/Scheffe's post hoc test or the Kruskal–Wallis/Mann–Whitney tests were employed. *P* values <0.05 were considered statistically significant.

## 3. Results

### 3.1. Lm-ME Reduced Nitric Oxide (NO) Production

**Figure 1:**
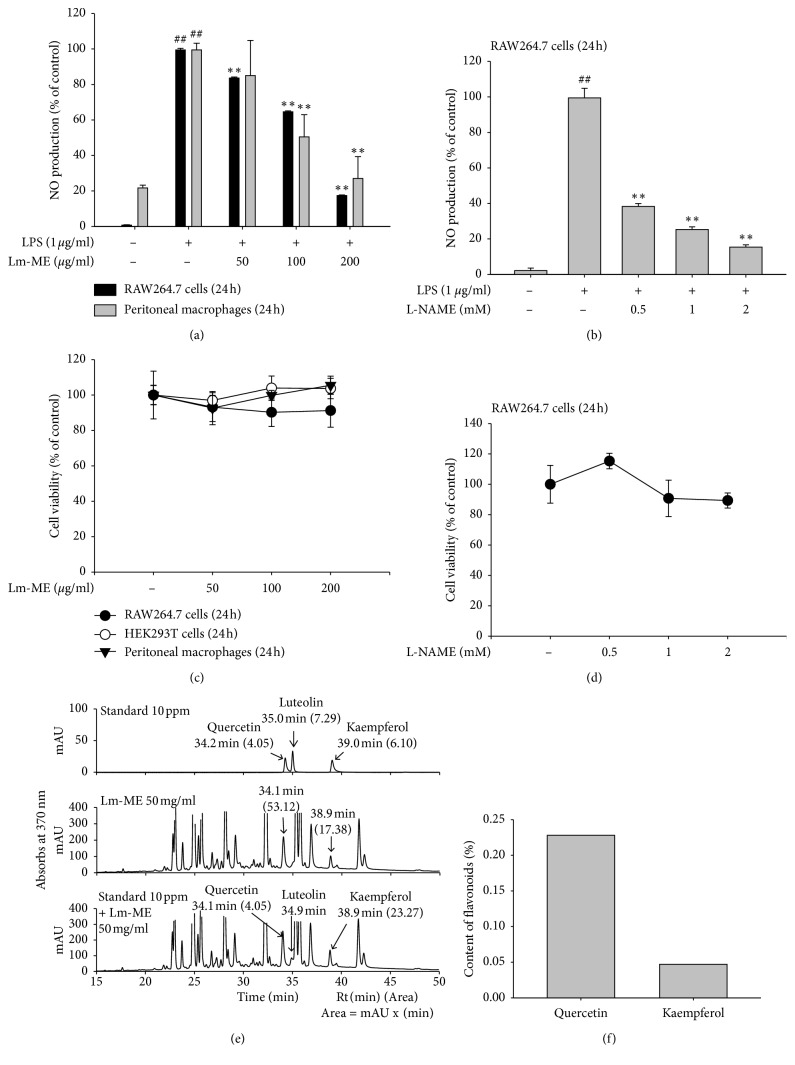
Effects of Lm-ME on NO production and cell viability. (a) Macrophage-like RAW264.7 cells and primary macrophage cells obtained from the peritoneal exudates of thioglycollate- (TG-) injected mice were pretreated with 50, 100, or 200 *μ*g/ml of Lm-ME for 30 min before treatment with LPS for 24 h NO production was measured using the cell culture supernatant. (b) RAW264.7 cells were pretreated with 0.5, 1, or 2 mM L-NAME and then treated with LPS. After 24 h incubation, NO collected from cell culture supernatants was measured by Griess solution. (c) RAW264.7 cells, HEK293T cells, and peritoneal macrophages were treated Lm-ME 50, 100, or 200 *μ*g/ml. After 24 h, cell viability was measured by MTT assay. (d) Cytotoxicity of L-NAME was determined by MTT assay. (e, f) Components of Lm-ME were analyzed by HPLC.

To figure out the inflammatory effects of Lm-ME, we first checked the inhibitory effect on NO production in LPS-induced RAW264.7 cells and peritoneal macrophages (Figure 1(a)). We used L-NAME as a positive control, because L-NAME exhibits selectivity for inhibition of NOS [26], and we say that it decreased NO production (Figure 1(b)). Importantly, Lm-ME (50–200 *μ*g/ml) did not show any cytotoxicity in RAW264.7 cells, HEK293T cells, or peritoneal macrophages by MTT assay (Figure 1(c)), so NO decreases were not due to cell death. Next, to verify the components of Lm-ME that might be responsible for anti-inflammatory effects, we performed HPLC analysis using flavonoids, including quercetin, kaempferol, and luteolin, because they are known to have anti-inflammatory effects [27]. Lm-ME contained 2.287 mg/g of quercetin and 0.477 mg/g of kaempferol (Figure 1(d)), implying that these flavonoids (quercetin and kaempferol) in Lm-ME suppressed LPS-induced NO production.

### 3.2. Anti-Inflammatory Effects of Lm-ME on Transcriptional Level

**Figure 2:**
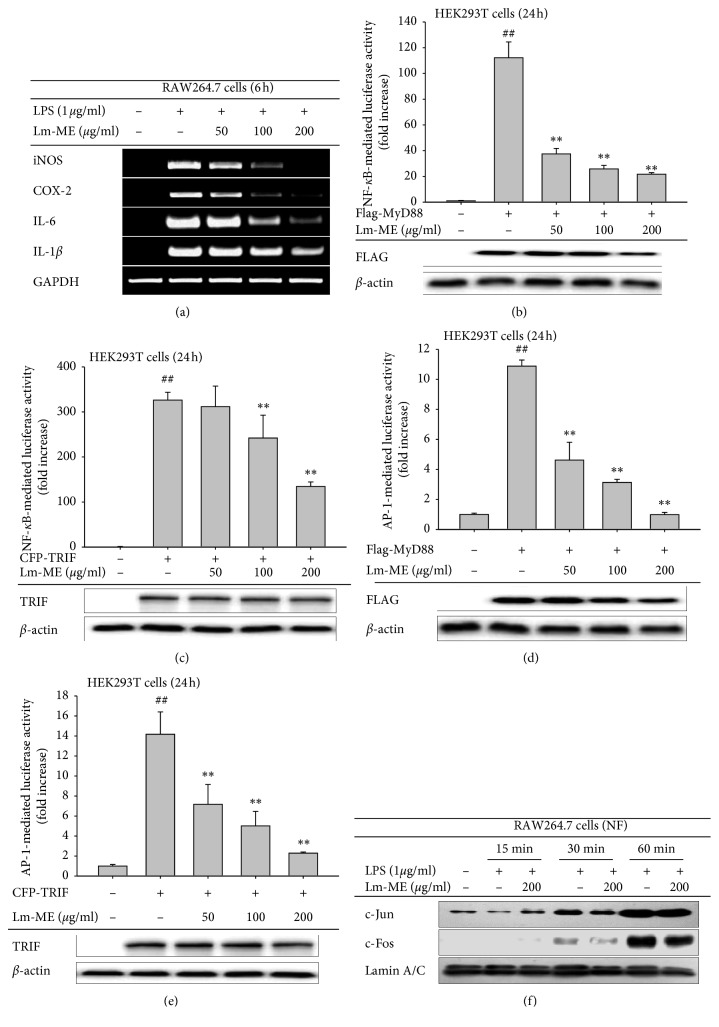
Anti-inflammatory effects of Lm-ME on mRNA expression and transcriptional activity. (a) RAW264.7 cells were pretreated with 50, 100, or 200 *μ*g/ml of Lm-ME and then treated with 1 *μ*g/ml of LPS. After 6 h, mRNA was extracted from the cells, and proinflammatory cytokines (iNOS, COX-2, IL-6, and IL-1*β*) were evaluated using semiquantitative RT-PCR. (b–e) In 24 wells, HEK293T cells were plated at 1.25 × 10^5^ cells per well. After 24 h, NF-*κ*B or AP-1 luciferase gene and *β*-galactosidase-expressing constructs were transfected into HEK293T cells along with either the MyD88 or TRIF gene. After 24 h incubation, 50, 100, or 200 *μ*g/ml of Lm-ME was added, and NF-*κ*B luciferase activity was measured by the luminometer. Total form of FLAG and TRIF was determined by western blotting analysis. (d) RAW 264.7 cells were pretreated with 200 *μ*g/ml of Lm-ME for 30 min and then exposed to 1 *μ*g/ml LPS for 15, 30, or 60 min. Using western blotting analysis, nuclear c-Jun, and c-Fos levels were determined.

Treatment of RAW264.7 cells with LPS can induce mRNA expression of proinflammatory molecules [28]. Accordingly, we investigated mRNA expression levels of proinflammatory genes. iNOS, COX-2, and IL-6 were strongly inhibited by 100 and 200 *μ*g/ml Lm-ME, and IL-1*β* was also slightly inhibited by Lm-ME (Figure 2(a)). In addition, we checked the transcriptional inhibitory effect of Lm-ME using a luciferase reporter gene assay. We transfected MyD88 or TRIF, which are key molecules in TLR4 signals, into macrophages with NF-*κ*B and AP-1 luciferase promoter genes [29]. Transfection of adaptor molecules (MyD88 and TRIF) in HEK293T cells mimicked the activation of RAW264.7 cells with LPS [30]. NF-*κ*B luciferase activity was decreased by Lm-ME in a dose-dependent manner in both MyD88 and TRIF-transfected conditions (Figure 2(b)). Moreover, AP-1 luciferase activity was decreased by Lm-ME in MyD88 and TRIF-transfected conditions (Figure 2(c)). Based on the luciferase assay results, we next explored whether Lm-ME downregulated nuclear translocation levels of c-Jun and c-Fos. At 30 and 60 min, the Lm-ME-treated group had lower c-Jun and c-Fos protein levels in the nucleus (Figure 2(d)). The protein levels of MyD88 (by FLAG) and TRIF in luciferase assay looked to be similar between all groups (Figures 2(b)–2(e)). In conclusion, based on luciferase activity and nuclear c-Jun and c-Fos results, Lm-ME can alleviate the LPS-induced signaling pathway.

### 3.3. Regulatory Mechanism of Lm-ME in NF-*κ*B and AP-1 Pathways

**Figure 3:**
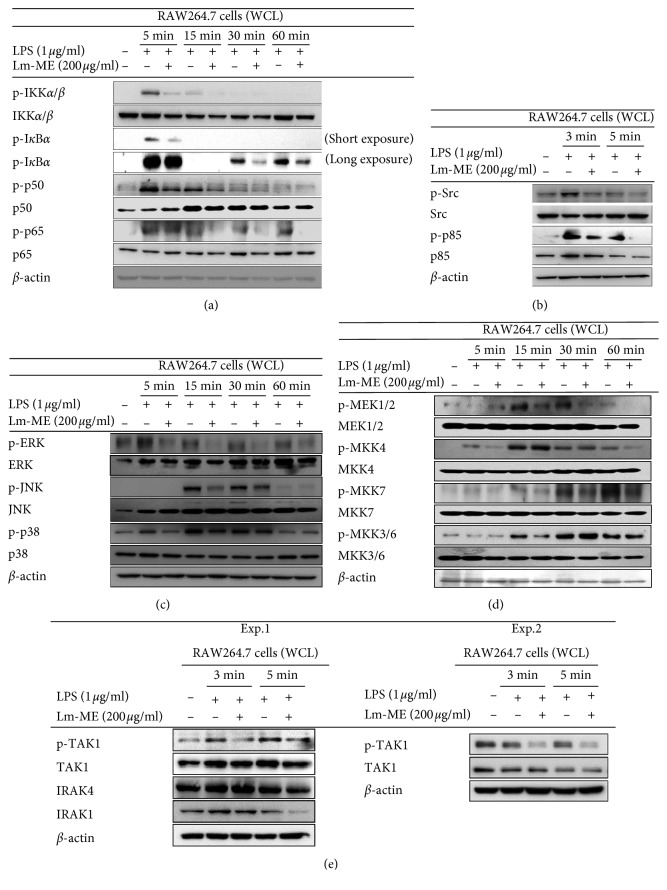
Inhibitory effects of Lm-ME on the NF-*κ*B and AP-1 pathways. (a) RAW264.7 cells were pretreated 200 *μ*g/ml of Lm-ME for 30 min, followed by 1 *μ*g/ml of LPS for the indicated times (5, 15, 30, or 60 min). Cell lysates were prepared and the phosphorylated and total forms of IKK*α*/*β*, p50, p65, and I*κ*B*α* were determined by western blotting analysis. (b) Lm-ME-pretreated RAW264.7 cells were exposed to LPS for the indicated times (3 or 5 min), and cell lysates were obtained. Phosphorylated and total forms of Src and p85 were checked by western blotting analysis. (c–e) RAW 264.7 cells were pretreated with 200 *μ*g/ml of Lm-ME and incubated with LPS for the indicated times (3, 5, 15, 30, or 60 min). Phosphorylated and total forms of ERK, JNK, p38, MEK1/2, MKK4, MKK3/6, and MKK7 or TAK1, IRAK4, and IRAK1 were determined by western blotting analysis.

Since proinflammatory cytokines (e.g., IL-6 and IL-1*β*) are related to the NF-*κ*B pathway, we expected Lm-ME to attenuate signal proteins passing through the NF-*κ*B pathway. To explore the effect of Lm-ME on NF-*κ*B signaling, we treated cells with Lm-ME at a concentration of 200 *μ*g/ml, because 200 *μ*g/ml of Lm-ME showed the highest NO inhibition without cytotoxicity (Figures 1(a) and 1(b)). When we checked western blotting in a time-dependent manner, the protein levels of phosphorylation of NF-*κ*B transcription subunits p50 and p65 were downregulated starting 5 and 15 min after treatment with 200 *μ*g/ml Lm-ME, and phospho-I*κ*B*α* clearly decreased starting after 5 min (Figure 3(a)). In LPS induction, activation of c-Src and ubiquitous Src tyrosine kinase is required for the NF-*κ*B pathway activation in macrophages [31]. To check responses over a short time period, we conducted western blotting after 3 and 5 min in LPS-treated RAW264.7 cells to determine the amount of phosphorylation of protein tyrosine kinase (Src) and phosphoinositide 3-kinases (p85). Both were suppressed by Lm-ME starting at 3 min (Figure 3(b)), implying that Src is a target protein of Lm-ME.

Previous data from the luciferase assay and nuclear fraction showed that Lm-ME can also attenuate the AP-1 pathway (Figures 2(c) and 2(d)). To find target molecules in AP-1 signaling, we conducted western blotting analysis to examine mitogen-activated protein kinases (MAPKs) including extracellular signal-regulated kinases (ERKs), c-Jun N-terminal kinases (JNKs), and p38. ERK was inhibited at all times (0–60 min), and JNK and p38 phosphorylation was decreased by Lm-ME at 15 min and at 5, 15, and 30 min, respectively (Figure 3(c)). Further, we checked signaling molecules upstream of mitogen-activated protein kinase kinase proteins (MAPKKs, e.g., MEK1/2, MKK4, MKK3/6, and MKK7). Phospho-MEK1/2, MKK3/6, and MKK7 decreased after 15 min, and phosphor-MKK4 decreased in 5 and 60 min (Figure 3(d)) after treatment with Lm-ME. MAPKKs were attenuated by Lm-ME, so we checked TAK1, IRAK1, and IRAK4 at earlier time points, because TAK1 is related to the AP-1 pathway and activates MAPKs [32]. Phospho-TAK1 decreased with Lm-ME treatment, but IRAK1 and IRAK4 were not affected by 200 *μ*g/ml Lm-ME (Figure 3(e)). Together, these data imply that TAK1 may be a target protein of Lm-ME.

### 3.4. Anti-Inflammatory Effects of Lm-Me by Targeting Src and TAK1 Kinases

**Figure 4:**
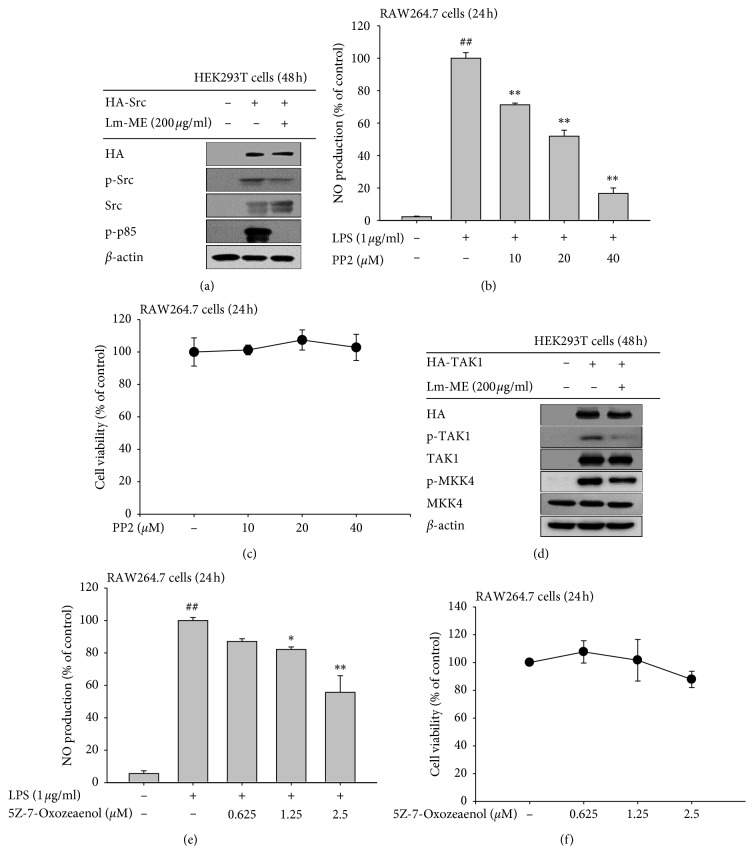
Overexpression of Src and TAK1 can be blocked by Lm-ME. (a) HEK293T cells were transfected with HA-Src constructs for 24 h and then divided according to treatment with Lm-ME. By western blotting analysis, total HA and Src and phosphorylated Src and p85 were detected. (b and e) RAW264.7 cells were pretreated with PP2 (an Src family inhibitor) and 5Z-7-oxozeaenol (a TAK1 inhibitor) at different concentrations. After 30 min, LPS was added for 24 h and the supernatant of RAW264.7 cells reacted with Griess reagents. NO production was measured by spectrophotometer. (c and f) The cytotoxicity of PP2 and 5Z-7-oxozeaenol were examined by MTT assay. (d) HEK293T cells were transfected with an HA-TAK1 expression construct for 24 h and also treated with Lm-ME for 24 h. Total HA, TAK1, and MKK4 and phosphorylated TAK1 and MKK4 were determined by western blotting analysis.

Based on previous western blotting results, we hypothesized that Src and TAK1 could be targeted by Lm-ME. To test this hypothesis, we utilized a strategy of overexpression of Src and TAK1 and determined whether autophosphorylation of Src and TAK1 and phosphorylation of downstream molecules decreased or not. First, we overexpressed HA-Src for 24 h in HEK293T cells and then treated the cells with 200 *μ*g/ml of Lm-ME for 24 h. c-Src is autophosphorylated on Tyr-416, a residue in the middle of the carboxyl terminus [33], so we checked the levels of phosphorylated Src and p-p85, which is phosphorylated by p-Src. Following treatment with Lm-ME, p-Src and p-p85 levels decreased (Figure 4(a)). Next, we sought to verify whether Src inhibition could block NO production. We used PP2, which was developed to inhibit Src family members [34]. RAW264.7 cells were pretreated with 10, 20, or 40 *μ*M of PP2 for 30 min and then treated with LPS for 24 h. As measured in the cell culture supernatant, PP2 blocked NO production without cytotoxicity (Figures 4(b) and 4(c)). In the AP-1 pathway, we assumed TAK1 could be the target protein of Lm-ME (Figure 3), so we overexpressed the TAK1 gene in HEK293T cells. TAK1 is also known as an autophosphorylated kinase within its activation loop [35]. We determined that phosphorylated TAK1 was activated and that Lm-ME could block autophosphorylation of TAK1. In addition, MKK4, a downstream molecule, was also inhibited by Lm-ME (Figure 4(d)). We further tested whether the TAK1 inhibitor 5Z-7-oxozeaenol could also inhibit NO production. As expected, 5Z-7-oxozeaenol reduced NO production without cytotoxicity (Figures 4(e) and 4(f)). Based on these findings, we conclude that Lm-ME can block phosphorylation of Src and TAK1.

### 3.5. Effect of Lm-ME on HCl/EtOH-Induced Acute Gastritis

**Figure 5:**
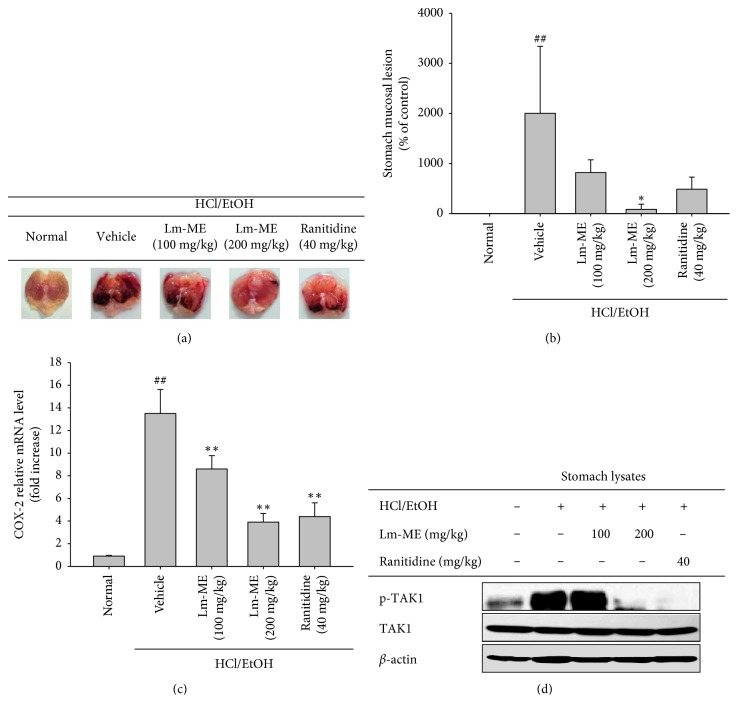
In vivo anti-inflammatory effects of Lm-ME. ICR mice were orally injected with 0, 100, or 200 mg/kg Lm-ME or 40 mg/kg of ranitidine 3 times over 2 days. Eight hours after the last oral injection, 300 *μ*l of 150 mM HCl/60% EtOH was orally administrated for 1 h. Stomachs were excised from the mice, and stomach lesions imaged (a). By using Image J software, stomach lesions induced by HCl/EtOH were measured (b). (c) mRNA expression levels of COX-2 from gastritis samples were measured by quantitative real-time PCR. (d) Total and phosphorylated TAK1 levels from gastritis mice were determined by western blotting analysis.

To investigate whether Lm-ME has pharmacological effects in vivo, the HCl/EtOH-induced gastritis animal model was employed under oral administration conditions. Since HCl/EtOH treatment causes injury and ulcer of the stomach, DAMPs could be released and seem to manage gastric inflammation [36, 37]. Interestingly, it has been reported that DAMP (e.g., ATP and high mobility group protein B1 (HMGB1))-mediated inflammation induces inflammation by interaction with TLRs (e.g., TLR4) and subsequent activation of the MyD88-dependent NF-*κ*B signaling pathway [36]. In the end, DAMPs can induce inflammatory cytokines such as IL-6, IL-1*β*, and TNF-*α* [38]. So, we used HCl/EtOH-induced gastritis model to check anti-inflammatory effect of Lm-ME. The 200 mg/kg Lm-ME group had the fewest stomach inflammatory blood lesions compared to 100 mg/kg Lm-ME and ranitidine (Figure 5(a)). In gastritis stomach samples, the level of COX-2 mRNA decreased after treatment with 200 mg/kg Lm-ME (Figure 5(c)). We next analyzed gastritis protein levels of TAK1 in its total and phosphorylated forms. Phosphorylated TAK1 was decreased by treatment with 200 mg/kg Lm-ME and 40 mg/kg ranitidine (Figure 5(d)). In conclusion, Lm-ME alleviated acute gastritis symptoms by inhibition of TAK1.

## 4. Discussion

The genus *Licania* displays the largest number of biological activities among Chrysobalanaceae species and is used widely in Venezuela for anti-inflammatory properties [39]. In Northeastern Brazil, *Licania* leaves have been used to treat diabetes, stomach aches, diarrhea, and dysentery [20]. However, the underlying anti-inflammatory mechanisms in Lm-ME in LPS-induced RAW264.7 cells and an HCl/EtOH-induced acute gastritis model have not previously been reported. Therefore, this study focused on the effects and molecular target proteins of Lm-ME to better illuminate anti-inflammatory molecular mechanisms.

When RAW264.7 cells and peritoneal macrophages are treated with LPS, they produce NO [40]. We observed that NO production was reduced by Lm-ME (Figure 1(a)) without any cytotoxicity in various LPS-stimulated cell types (Figure 1(b)). These results indicate that Lm-ME can have anti-inflammatory effects on macrophage-like RAW264.7 cells and peritoneal macrophages. Lm-ME significantly decreased mRNA levels of proinflammatory cytokines such as iNOS, COX-2, IL-6, and IL-1*β* in LPS-induced RAW264.7 cells (Figure 2(a)). These proinflammatory cytokines are related to inflammatory pain and disease [16]. IL-1*β* cytokines and IL-6 are related to rheumatologic autoimmune diseases, and many treatment strategies have targeted cytokine production. In addition, NO release and iNOS expression can be caused by osteoarthritis and systemic lupus erythematosus (SLE), as well as various types of intestinal inflammation [41]. We found that the HCl/EtOH-induced acute gastritis model of damage-associated inflammatory disease was palliated by Lm-ME, which reduced the mRNA levels of COX-2 and protein levels of phosphorylated TAK1 (Figures 5(c) and 5(d)). The regulatory effects of Lm-ME on NO production and expression of other cytokines suggest the possibility that Lm-ME can be used as an effective alternative remedy in inflammatory-related disorders.

Flavonoids are polyphenolic compounds that occur ubiquitously in plants, and they have been found to have antioxidant, antiplatelet, antiviral, antiulcerogenic, antimicrobial, antihypertensive, and anti-inflammatory activities. Kaempferol, quercetin, and myricetin are well known flavonols that have a 3-hydroxyflavone backbone and anti-inflammatory effects [42–45]. Accordingly, we checked the active components of Lm-ME and found ample quercetin and kaempferol (Figures 1(e) and 1(f)). Quercetin and kaempferol are reported to reduce iNOS, COX-2, reactive c-protein, and NF-*κ*B pathway signaling molecules [43]. Our findings are in agreement with noted inhibition effects of Lm-ME on mRNA production (Figure 2(a)), NF-*κ*B luciferase activity (Figure 2(b)), and active forms of NF-*κ*B transcription factors p50 and p65 (Figure 3(a)).

Interestingly, Lm-ME targeted Src tyrosine kinase by blocking autophosphorylation of Src and suppressing downstream signaling molecules PI3K, IKK*α*/*β*, I*κ*B*α*, p50, and p65 (Figures 3(a) and 3(b)). Central role of Src in controlling inflammatory signaling cascades for NF-*κ*B activation has been previously reported [46, 47]. Due to the functional response of Src in inflammatory signaling, inflammation-regulatory activities of Src including cytokine production, migration of macrophages, and inflammatory mediator production have been also found in LPS-treated macrophages [48, 49]. Several compounds or extracts with Src inhibitory property were also proved as anti-inflammatory reagents [14, 50–53]. In the AP-1 pathway, Lm-ME suppressed activation of only TAK1 and not IRAK1 and IRAK4 (Figure 3(e)). IRAK is membrane associated as part of a complex (IRAK-TRAF6-TAK1-TAB2-TAB3). The important step in inflammation signaling requires IRAK1 to be consecutively phosphorylated on threonine 209, ubiquitinated and degraded. This step allows the TRAF6-TAK1-TAB2-TAB3 complex to translocate from the membrane to the cytosol, and degradation is a necessary step in activation of the TAK1-dependent pathway [54, 55]. TAK1 plays roles in both NF-*κ*B and AP-1 pathways, which phosphorylate and activate IKK and MKK6 (an upstream molecule of JNK and p38) [56]. Indeed, it was reported that TAK1 inhibitor 5Z-7-oxozeaenol can prevent picryl chloride-induced ear swelling [57]. Some anti-inflammatory plants or compounds such as *Momordica charantia* and torilin were found to suppress TAK1 as a pharmacological target [12, 58], implying that this enzyme plays a positive role in inflammatory responses. Lm-ME suppressed the activation of TAK1 in both LPS/TLR4-activated macrophages (Figure 3(e)) and in HCl/EtOH-induced gastritis symptom (Figure 5(d)), so it is possible that the observed downregulation of IKK*α*/*β* resulted from both Src and TAK1 inhibition.

**Figure 6:**
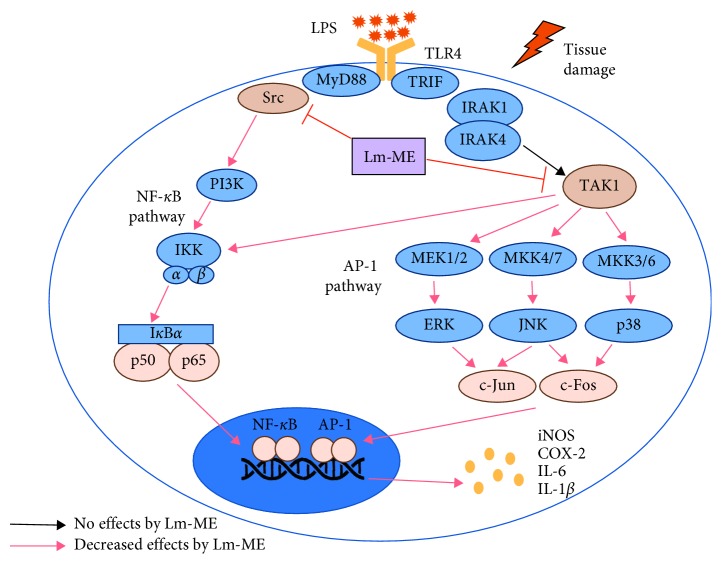
Anti-inflammatory mechanisms of Lm-ME. Lm-ME targeted Src and TAK1 kinases in inflammatory NF-*κ*B and AP-1 pathways. Consequently, the activation of transcription factors was blocked, and various inflammatory cytokines and mediators were reduced.

In conclusion, we found that Lm-ME exerted anti-inflammatory effects by targeting Src- and TAK1-dependent pathways. Lm-ME decreased NO production and transcription of proinflammatory cytokines in vitro and also reduced stomach lesions, levels of COX-2 mRNA, and phosphorylated TAK1 in an HCl/EtOH-induced gastritis model in vivo. Based on these findings, *L. macrocarpa* Cuatrec could be a useful herbal medicine for the prevention of Src- and TAK1-related inflammatory diseases (Figure 6).

## Figures and Tables

**Table 1:** Sequences of primers used in RT-PCR analysis.

Gene	Sequences (5′ ⟶ 3′)
iNOS	
Forward	5′-CCCTTCCGAAGTTTCTGGCAGCAGC−3′
Reverse	5′-GGCTGTCAGAGCCTCGTGGCTTTGG−3′
COX-2	
Forward	5′-CACTACATCCTGACCCACTT−3′
Reverse	5′-ATGCTCCTGCTTGAGTATGT−3′
IL-6	
Forward	5′-GTACTCCAGAAGACCAGAGG−3′
Reverse	5′-TGCTGGTGACAACCACGGCC−3′
IL-1*β*	
Forward	5′-CAGGATGAGGACATGAGCACC−3′
Reverse	5′-CTCTGCAGACTCAAACTCCAC−3′
GAPDH	
Forward	5′-CACTCACGGCAAATTCAACGGCAC−3′
Reverse	5′-GACTCCACGACATACTCAGCAC−3′

**Table 2:** Sequences of primers used in real-time analysis.

Gene	Sequences (5′ ⟶ 3′)
COX-2	
Forward	5′-AAGGTGAGAAGCAATGCAGC−3′
Reverse	5′-CCACTCAGGGAGTTCTCTCT−3′
GAPDH	
Forward	5′-CAATGAATACGGCTACAGCAAC−3′
Reverse	5′-AGGGAGATGCTCAGTGTTGG−3′

## Data Availability

The data used to support the findings of this study are available from the corresponding author upon request.
